# External Beam Radiotherapy for Head and Neck Cancers Is Associated with Increased Variability in Retinal Vascular Oxygenation

**DOI:** 10.1371/journal.pone.0069657

**Published:** 2013-08-06

**Authors:** Daniel S. Higginson, Alok Sahgal, Michael V. Lawrence, Sarah Moyer, Mihaela Stefanescu, Adam K. Willson, Bahjat Qaqish, Adam Zanation, Lawrence B. Marks, Seema Garg, Bhishamjit S. Chera

**Affiliations:** 1 Department of Radiation Oncology, Memorial Sloan-Kettering Cancer Center, New York, New York, United States of America; 2 Department of Ophthalmology, University of North Carolina at Chapel Hill, School of Medicine, Chapel Hill, North Carolina, United States of America; 3 Department of Radiation Oncology, University of North Carolina at Chapel Hill, School of Medicine, Chapel Hill, North Carolina, United States of America; 4 Department of Biostatistics, University of North Carolina at Chapel Hill, Gillings School of Global Public Health, Chapel Hill, North Carolina, United States of America; 5 Department of Otolaryngology, University of North Carolina at Chapel Hill, Chapel Hill, North Carolina, United States of America; Northwestern University Feinberg School of Medicine, United States of America

## Abstract

**Background:**

Radiation retinopathy is a possible post-treatment complication of radiation therapy. The pathophysiologic mechanism is hypothesized to be microvascular in origin, but evidence is limited. In an effort to study retinal oxygenation in these patients, we herein evaluate the repeatability and variability of retinal oximetry measurements in subjects who had previously received radiation and make comparisons to a cohort of unirradiated subjects.

**Methods:**

Using retinal oximetry, a non-invasive imaging modality, we performed *in vivo* measurements of arteriole (SaO_2_) and venule SO_2_ (SvO_2_) in subjects (n = 9, 18 retinas) who had received incidental radiation to their retinas (≥ 45 Gy to one retina) and in healthy subjects (n = 20, 40 retinas). A total of 1367 SO_2_ observations on 593 vessels in 29 persons were analyzed to assess three sources of variance in vessel SO_2_: 1) variance in repeated measurements of the same vessel (“repeatability”), 2) variance in different vessels within the same subject (“within-subject variability”), and 3) variance between subjects (“between-subject variability”).

**Results:**

Retinal oximetry measurements were highly repeatable in both irradiated patients and unirradiated subjects. The within-subject variability of SvO_2_ and SaO_2_ measurements constituted the highest component of variance in both groups and was significantly higher in venules vs. arterioles (relative effect size 1.8, p<0.001) and in irradiated subjects vs. unirradiated subjects (relative effect size 1.6, p<0.001).

**Conclusions:**

Retinal oximetry is a highly repeatable technology and can be reliably used to study vascular oxygenation in irradiated subjects. Different vessels within the same subject exhibit a high degree of variability, suggesting that pooled analyses of multiple vessels are most likely to be informative of regional retinal oxygenation. Finally, irradiated subjects exhibited significantly higher within-subject variability in SO_2_ measurements, suggesting that radiation may cause regional alterations in retinal oxygen delivery and/or metabolism.

## Introduction

Radiation therapy is an important treatment modality for tumors of the sinonasal region, nasopharynx, and brain. Often the retina is unavoidably and incidentally irradiated. Radiation retinopathy is a potential long-term complication, particularly when the retina receives doses greater than 45 Gy [1]–[5].

Serial observational studies, using fluorescein angiography and fundus photography, have demonstrated early vascular changes such as microaneurysms, capillary obliteration, and telangiectasias, followed by exudate formation, macular edema, and neovascularization in some cases [6]–[8].

One leading hypothesis for the mechanism of radiation retinopathy is microvascular injury, which results in retinal hypoxia, neuronal cell loss, and ultimately visual impairment, analogous to diabetic retinopathy [6], [9], [10]. Direct evidence of ischemia or oxygenation changes in the retina after radiation retinopathy is limited. Specifically *in vivo* observations of early changes in retinal oxygenation physiology are not known.

Retinal oximetry is an emerging non-invasive retinal imaging modality for measuring *in vivo* changes in retinal vessel oxygen saturation (SO_2_). The technical aspects of retinal oximetry have been previously described in the literature [11]–[13]. Spectrophotometric retinal oximetry measures intravascular oxygen saturation by employing differences in light absorption characteristics between oxygenated and deoxygenated hemoglobin. Retinal arterial (SaO_2_) and venular oxygenation saturation (SvO_2_) can be displayed on conventional fundus camera images by color mapping of SO_2_ values along the length of selected vessels, providing qualitative and quantitative measures of regional retina vascular oxygenation.

The method has previously been shown as reproducible and sensitive to changes in O2 concentration of inspired gas [13]. Diabetic subjects with retinopathy have been evaluated with retinal oximetry and increases in SvO2 and decreases in SaO_2_-SvO_2_ differences (i.e. oxygen extraction) have been observed in proportion to the severity of retinopathy [14], [15]. It has been used to investigate the pathophysiology of retinal disorders hypothesized to be vascular in etiology or related to oxygenation, such as open angle glaucoma, diabetic retinopathy, cyanotic cardiac defects, and central retinal artery occlusion [14]–[18].

We hypothesize that retinal oximetry measurements are reproducible in irradiated retinas and radiation causes changes in retinal oxygenation that are detectable with retinal oximetry. To study this hypothesis we performed retinal oximetry in subjects who incidentally received high doses of radiation (>45 Gy) to their retinas for tumors near the eyes (irradiated cohort) and in normal subjects (un-irradiated cohort). We assessed the reproducibility of retinal oximetry and the variability of retinal oxygenation. We analyzed three components of variance using a random effects model: 1) variance between repeated measurements of the same vessel (“repeatability”), 2) variance between different vessels within the same subject (“within-subject variability”), and 3) variance between subjects (“between-subject variability”). We consider potential factors that may influence the components of variance model, including vessel length and width, vessel type (arteriole vs. venule), and cohort (irradiated subjects vs. unirradiated subjects).

## Methods

### Subject Selection

All analyzed subjects were enrolled on one of two protocols approved by an institutional review board at the University of North Carolina (UNC) Lineberger Comprehensive Cancer Center. The irradiated subjects (N = 9) were enrolled on the LCCC 1019 “Changes in Regional Retinal Oxygen Extraction and Function after Radiation Therapy.” The major inclusion criteria for this study were 1) >18 years of age and 2) subjects receiving an average dose of >45 Gy to at least one quadrant of one retina. Subjects with recurrent/persistent disease or subjects with confounding co-morbidities (e.g. diabetes, glaucoma) were excluded. The unirradiated subjects (N = 20) were enrolled in an internal study designed to acquire normative data in healthy subjects (LCCC 10–2175).

### Radiation Dosimetry for the Irradiated Cohort

Subjects were simulated for radiation treatment planning with an aquaplast head cast for immobilization and a 100 kV CT Big Bore scanner with 3mm axial slicing. Radiation plans were formulated with the PlanUNC (PLUNC) treatment planning system (UNC, Chapel Hill). To calculate retinal doses, each globe was manually contoured and the retina volume was defined as the space between the periphery of the globe and a 5 mm concentric contraction of this volume. The % volume of retina receiving >45 Gy and the maximum and mean doses to each retina were calculated. All of the subjects in the irradiated cohort were treated with intensity modulated radiotherapy (IMRT), which creates highly conformal radiation dose distributions with steep dose gradients allowing for sparing of the optic apparatus. It is standard practice to maximally spare one retina from high doses of radiation. However, the retina adjacent to the tumor may unavoidably receive >45 Gy. The steep dose gradient created by IMRT means that typically only the medial portion of one retina received ≥45 Gy, potentially leading to geographically varied effects on the retina in the same subject.

### Visual Acuity

Irradiated subjects' visual acuity was measured using an ESV-3000 ETDRS testing device with standardized back lighting placed 4 meters in front of the subject [19]. Right and left eyes were assessed separately with pupils undilated. The control subjects were assessed using Snellen visual acuity charts. Subjects were categorized into groups according to their eye with the worst acuity (normal: 20/25 or better; near normal: 20/32–20/63; moderate or worse: <20/80) [20].

### Retinal Oximetry

All images were acquired by trained retinal photographers using the Oxymap Retinal Oximeter (Reykjavik, Iceland) mounted on a Topcon TRC50-VT fundus camera (Topcon Co, Tokyo, Japan). For each subject, the images were centered on the optic disc with a 50-degree field of view. Prior to image capture, subjects' pupils were dilated with tropicamide (1%) and phenylephrine hydrochloride (2.5%) eye drops. All oximetry data was obtained with identical lighting conditions (door closed, lights off). For each image, the optic disc was centered and a 50° field of view was obtained. The subjects' gaze was directed straight ahead to minimize angle-of-gaze-dependent effects [21]. The sensor on the imaging camera is 1600×1200 pixels.

The relationship between absorbance and SO_2_ was defined using calibration parameters provided by Oxymap (SO_2_  = −0.953*ODR +1.16, ODR = optical density ratio). Absorbance measurements are obtained at two wavelengths: 570 nm and 600 nm. A minimum pixel width of 8 was chosen as the threshold for analysis as recommended by Oxymap (personal communication). All vessels above the threshold of 8 pixels were included in the analysis.

An SO_2_ value for a given vessel (arteriole, SaO_2_ and venule, SvO_2_) represents a weighted average of SO_2_ along the length of a vessel. Vessels were analyzed from the edge of the optic disc to the periphery of the captured image. Where two vessels crossed, a small 3–4 pixel area of exclusion was used, per Oxymap recommendations. All branches of an analyzed vessel were included in the weighted average, provided they were larger than 8 pixels in diameter.

For the first five subjects in each cohort (irradiated group and unirradiated control group), five repeated oximetry images were captured within a few minutes of each other. Each vessel was analyzed on each of the five repeated oximetry images and compared for the repeatability analysis. After an interim analysis was done and high repeatability for both the irradiated and unirradiated cohorts was found, only one image was analyzed for subjects 6–9 (irradiated cohort) and 6–20 (control cohort) for use in the remaining components of variance analysis.

### Statistics

A components of variance model was used to allow for modeling the three levels of variation: 1) variance between repeated measurements of the same vessel (“repeatability”), 2) variance between different vessels within the same subject (“within-subject variability”), and 3) variance between subjects (“between-subject variability”). Each of the three variance components was modeled as a log-linear regression on covariates. Maximum-likelihood estimates were obtained, and likelihood ratio tests were used to compute p-values. Standard errors were based on the Fisher information matrix. The analysis was done in R, version 2.14. A total of 1367 SO_2_ observations on 593 vessels in 29 subjects were analyzed and fit to the model.

## Results

### Subject Characteristics

Nine irradiated and 20 unirradiated subjects underwent retinal oximetry (Table 1). There were more males and more heavy smokers in the irradiated cohort, but the differences were not significant by Fisher's exact test (p = 0.40 and 0.57, respectively). None of the subjects exhibited severe deficits in ETDRS visual acuity testing, suggesting that the SO_2_ measurements in the irradiated cohort were obtained prior to clinically symptomatic retinopathy and any observed changes in retinal oxygenation would represent early subclinical changes (Table 1). Among the irradiated subjects, the mean dose to the retina ranged from 9.1 to 54.0 Gy (see Table 2). Each subject in the irradiated cohort received ≥45 Gy to a significant portion of at least one retina (range, 19.5 to 94.0%).

**Table 1:** Subject characteristics.

Characteristic	Irradiated Subjects	Unirradiated Subjects
	N = 9 No. (%)	N = 20
	No. (%)	No. (%)
Age (mean, range)	54 (35–73)	52 (26–71)
Sex		
Male	4 (44%)	5 (25%)
Female	5 (56%)	15 (75%)
Race		
Caucasian	7 (78%)	16 (80%)
African-American	1 (11%)	2 (10%)
Hispanic	1 (11%)	1 (5%)
Asian	0 (0%)	1 (5%)
Visual acuity class		
Normal (20/12.5 – 20/25)	4 (44%)	16 (80%)
Near Normal (20/32 – 20/63)	5 (56%)	2 (10%)
Moderate or worse (<20/80)	0 (0%)	0 (0%)
Missing Data	0 (0%)	2 (10%)
Smoking history		
Never	3 (33%)	15 (75%)
Light (<15 pack-years)	4 (44%)	2 (10%)
Heavy (>15 pack-years)	2 (22%)	2 (10%)
Missing Data	0 (0%)	1 (5%)
Vascular co-morbidities		
Peripheral vascular disease	0 (0%)	0 (0%)
Hypertension	3 (33%)	3 (15%)
Stroke	0 (0%)	0 (0%)
Coronary artery disease	0 (0%)	0 (0%)
Hyperlipidemia	1 (11%)	4 (20%)
Missing Data	0 (0%)	1 (5%)

**Table 2:** Disease characteristics and radiation dose to the retina of irradiated subjects.

					OD			OS		
No.	Disease Site	Histology	Stage	Prescription Dose†	Mean Dose (Gy)*	Max Dose (Gy)*	% of retina receiving > 45 Gy	Mean Dose*	Max Dose*	% of retina recei ving > 45 Gy
1	L ethmoid sinus	Adenocarci noma	T4aN0M0	62.4 Gy/1.2 Gy BID	18.4	44.1	0	29.9	54.8	19.5
2	R maxillary sinus	Adenoid cystic carcinoma	T4bN0M0	62 Gy/2 Gy	44.4	64.5	51.0	3.58	17.8	0
3	Nasopharynx	Nasopharyngeal carcinoma, WHO type III	T4N2cM0	68 Gy/2 Gy	55.6	69.4	85.2	35.1	54.9	12.2
4	L maxillary sinus	Squamous cell carcinoma	T4bN0M0	70.2 Gy/1.8 Gy	8.23	35	0	59.7	74	93.8
5	R ethmoid sinus	Sinonasal undifferentiated carcinoma	T4aN0M0	63 Gy/2 Gy	47.6	60.2	67.9	11.8	40.7	0
6	R ethmoid sinus	Sinonasal undifferentiated carcinoma	T2N0M0	59.4 Gy /1.8 Gy	30.0	60.9	22.6	16.2	50.7	5.1
7	L maxillary and ethmoid sinuses	Neuroendocrine carcinoma	T4bN0M0	60 Gy/2 Gy	23.7	51	1.5	58.6	80.3	87.8
8	R maxillary sinus	Squamous cell carcinoma	T2N0M0	59.4 Gy/1.8 Gy	31.3	63.3	31.0	11.0	51.0	1.1
9	R nasal vestibule	Squamous cell carcinoma	T4aN0M0	73.2 Gy/2 Gy/1.2 Gy BID	40.1	65.7	38.4	36.9	65	32.1

† Listed as total dose/dose per fraction. BID: twice a day fractions.

* Doses to the retina, as defined in the methods section.

### Influence of Vessel Width and Length on SO_2_ Values

We first evaluated whether SO_2_ measurements of a given vessel (SaO_2_ and SvO_2_) was influenced by the length or the width of that vessel, which can vary anatomically. SaO_2_ did not correlate with either vessel length or width. As seen previously [22]. SvO_2_ measurements did vary linearly with vessel width in both the irradiated and unirradiated cohorts, but not length. Venule vessel width was therefore incorporated into our components of variability analysis, but did not by itself independently influence either same vessel repeatability or within-subject variability (Table 3).

**Table 3:** Factors influencing variability in vascular SO_2_ by retinal oximetry.

	Co-variate	SD of SO_2_ Measurements	Relative Effect Size*	p-value
Between-subject variability	Un-IR group vs. IR group	2.23% vs. 3.63%	1.63	0.14
Between- vessel variability (within the same patient)	Un-IR vs. IR group	5.06% vs. 8.19%	1.62†	<0.001
	Arterioles vs. venules	2.78% vs. 5.06%	0.55‡	<0.001
	Vessel width	–	0.993	0.71
Between- vessel variability (within the same patient)	Un-IR vs. IR group			
	Arterioles vs. venules			
	Vessel width			
Same vessel variability (repeatability of measurement)	Un-IR vs. IR group	2.39% vs. 2.48%	1.04	0.41
	Arterioles vs. venules	1.70% vs. 2.39%	0.71§	<0.001
	Vessel width	–	1.01	0.27

SD: Standard deviation; Un-IR: unirradiated group; IR: irradiated group.

* Standard deviation of SO_2_ measurements in one patient co-variate category divided by another co-variate category.

† Between-vessel SD in the irradiated group is 1.62 times the unirradiated group for vessels of the same type and width.

‡ Between-vessel SD for arterioles is 0.55 that of venules for vessels of same width and in the same group of patients.

§ The SD of repeated measurements of the same vessel is 0.71 fold smaller for arterioles than for venules in vessels of the same width and in the same group of patients.

### Repeatability of Retinal Oximetry Measurements

For the first five subjects in both the irradiated and the unirradiated cohorts, five repeated measurements of each arteriole and venule were obtained (197 vessels, 985 measurements). The standard deviations within repeated same-vessel SO_2_ measurements were small: 2.39–2.50% for venules and 1.70–1.77% for arterioles (Table 4). These figures constituted only 7–18% of the total variability in all measurements (see Table 4). Venules exhibited significantly worse repeatability than arterioles (p<0.001, Table 3) in all subjects even with adjustment for vessel width, but in absolute terms the repeatability was comparable to other reports [22]. There were no differences in same vessel repeatability between the unirradiated and irradiated groups (Table 4).

**Table 4:** Components of variability in vascular SO_2_ measurements by retinal oximetry.

		Components of Variability in Absolute Numbers				Components of Variability in Terms of % of Total Variability			
Group	Vessel Type	Between Subject SD (%SO_2_) *	Between Vessel SD (%SO_2_) †	Same Vessel SD (%SO_2_) ‡	Total SD (%SO_2_)§	Between Subject SD (%)*	Between Vessel SD (%)†	Same Vessel SD (%)‡	Total Variability (%)§
Unirradia ted group	Venules	2.23%	5.06%	2.39%	6.02%	14%	71%	16%	100%
	Arterioles	2.23	2.79	1.70	3.95	32	50	18	100
Irradiated group	Venules	3.63	8.19	2.50	9.30	15	78	7	100
	Arterioles	3.63	4.52	1.77	6.06	36	56	9	100

SD: standard deviation.

* Variability in SO_2_ measurements between subjects within subject group and vessel types subgroups.

† Variability in SO_2_ measurements between vessels in the same subject.

‡ Variability in repeated SO_2_ measurements of the same vessel in the same subject.

§ Root-sum-square of the between-subject, between-vessel, and same-vessel SD components.

### Within-subject Variability in SO_2_ Values

When we analyzed the variability of SO_2_ measurements of different retinal vessels within the same subject, this constituted the highest proportion of variability for both groups and for both arterioles and venules (50–78% of all variability, Table 4). Variability amongst within-subject venules was significantly higher than arterioles (p<0.001, Table 3). For instance, for the irradiated subjects the standard deviation of all retinal venules was 8.2% vs. 4.5% for arterioles (p<0.001, Table 4).

**Figure 1:**
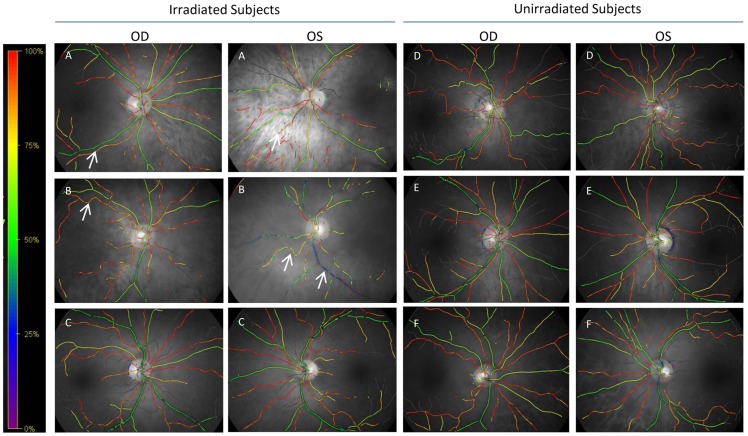
Representative qualitative retinal oximetry images of retinas from 3 irradiated (A–C) and 3 unirradiated (D–F) subjects from the two cohorts. OD: Right eye. OS: Left eye. A color gradient with corresponding SO_2_ values is provided at the left of the images. The green/yellow vessels are venules while the red/orange vessels are arterioles. Some of the irradiated subjects exhibited highly variable SO_2_ values in venules (see arrow to blue venule in patient B, OS) or arterioles (see arrows in patient B, OD and patient A, OD and OS). Not all irradiated subjects exhibited this marked variability in qualitative SO_2_ measurement (Subject C). Unirradiated subjects had less within-subject variability (note the more consistent green venules and red arterioles in patients D–F).

We observed significantly higher variability in irradiated subjects compared to un-irradiated subjects (Tables 3 and 4) with a relative effect size of 1.62 (p<0.001). The variability in irradiated venules was 8.2% compared to 5.1% in unirradiated venules. On visual inspection of the retinal oximetry images, there was a higher degree of uniformity of vascular oxygen saturation values in the unirradiated subjects (Figure 1, Panels D–F) compared to the irradiated subjects (Figure 1, Panels A–C).

### Between-subject Variability in SO_2_ Values

Next we analyzed variability in SO_2_ values between subjects within the two cohorts, which constituted 14–36% of the total variability (Table 4). There was no significant difference between the irradiated and unirradiated groups (Table 3).

## Discussion

The present study demonstrates that retinal oximetry measurements of SO_2_ are reproducible in irradiated subjects. We observed a higher degree of variability in venules as compared to arterioles (Table 3). Furthermore, there was greater variability in SO_2_ measurements in the irradiated cohort as compared to the unirradiated cohort (relative effect size of 1.62, p<0.001, Table 3). Stated another way, subjects that had had radiation exhibited more variability in their SaO_2_ and SvO_2_ measurements per vessel as compared to subjects in the unirradiated cohort. This finding suggests that the metabolism of oxygen in the retina may be altered after radiation therapy.

Furthermore, because the variability amongst different vessels within the same subject was high, any one single vessel by itself may not accurately depict retinal oxygenation throughout the retina, and thus in future studies pooled analyses of multiple vessels may be more appropriate. Variability between subjects within the same cohort was not significantly different. This observation suggests that any potential effect of radiation on retinal oxygenation was similar among the 9 subjects that received incidental irradiation of their retinas.

Our observations demonstrate that radiation appears to be associated with increases in the variation in SO_2_ values within a given subject. Since most of the irradiated subjects had non-uniform exposure of their retina to radiation (because of the steep dose gradients created by intensity modulated radiotherapy), perhaps the increased variability is consistent with the hypothesis that radiation can effect oxygenation of the retina. Additional work is needed to better relate the spatial variations in the effects seen to the associated radiation dose distribution.

The present work suggests that retinal oximetry is a promising approach to better study radiation-associated retinopathy. The degree of variability between subjects (see in both cohorts) is likely physiologic as known technical sources of variability were minimized in this study [21] and measurements were highly repeatable. This suggests that in future work, rather than simply comparing irradiated vs. unirradiated patients in terms of absolute SO_2_ (which can be variable physiologically), oximetry studies of irradiated patients are best performed with matched pre- and post-radiotherapy images or with intra-patient comparison of heavily irradiated versus unirradiated retinal regions.

The mechanism of radiation retinopathy has been proposed to be analogous to diabetic retinopathy, involving primarily microvascular injury leading to chronic ischemia and neuronal cell loss over time and in advanced cases, neovascularization. However, direct evidence of oxygenation alterations in irradiated patients is limited. We herein have reported the first *in vivo* evidence of altered retinal oxygenation after radiotherapy in albeit small cohort of subjects with correctable visual acuity. These observations of increased SO_2_ variability before symptomatic radiation retinopathy may represent early precursory evidence of retinal damage from radiation therapy.

This study does have certain inherent limitations. The subjects were analyzed after radiation had already been delivered and thus the relationship between these vascular changes and radiation may be correlative rather than causative. Future work will examine the relationship between regional retinal radiation dose and SO_2_ values within those regions, and prospectively follow subjects with retinal oximetry before and after radiation.
